# Supplementary material for: Antibiotic prescription patterns and associated symptoms in children living with HIV at Arthur Davison Children’s Hospital in Ndola, Zambia
Source: PLoS One. 2025 Mar 5;20(3):e0316735. doi: 10.1371/journal.pone.0316735 (PMC11882085; doi:10.1371/journal.pone.0316735)
Supplement: Attachment — Submitted filename: PONE-D-24-01009 (1).pdf [file pone.0316735.s002.pdf]

PLOS ONE

Common clinical presentations associated with antibiotic use in HIV-infected children at a large referral Children's Hospital in Northern Zambia.

--Manuscript Draft--

**Manuscript Number:**	PONE-D-24-01009
**Article Type:**	Research Article
**Full Title:**	Common clinical presentations associated with antibiotic use in HIV-infected children at a large referral Children's Hospital in Northern Zambia.
**Short Title:**	Clinical presentations associated with antibiotic use in HIV-infected children in Zambia
**Corresponding Author:**	Victor Daka, MSc<br>The Copperbelt University School of Medicine<br>Ndola, Copperbelt ZAMBIA
**Keywords:**	Antibiotics, ART, HIV, Children, Clinical presentation
**Abstract:**	Background Children with HIV infection are disproportionately susceptible to bacterial infections. Despite the availability of anti-bacterial agents to manage HIV children with bacterial infections, the indiscriminate administration of antibiotics still occurs at health facilities which could lead to antimicrobial resistance. Objectives This study aimed to determine common antibiotics administered and associated clinical presentation in children at Arthur Davison Children's Hospital (ADCH) ART (antiretroviral therapy) clinic in Ndola, Zambia. Methods This was a retrospective analysis of prescribing patterns of routinely collected data at ADCH. Complete enumeration of files for all children attending ART care at ADCH from January 2011 to October 2022 was conducted. Children diagnosed with HIV before the age of 5, actively attending ART clinic identified by SmartCare software and who had taken ART for 3 months were eligible. Results There were 132 children included in the study and of these, 37.9% (50/132) presented with symptomatic complaints with the most common clinical presentation being cough (70%) followed by fever (34%) and diarrhoea (30%). A larger proportion of children were on abacavir/lamivudine/dolutegravir (ABC/3TC/DTG) (62.1%) for the ART regimen of choice. Child taking the abacavir/lamivudine/dolutegravir (ABC/3TC/DTG) regimen more frequently had complaints (48.8%) than those on TAF/3TC/DTG (21.0%) or TDF/3TC/DTG regimen (18.2%) (p=0.006). Conclusions Common clinical presentations showed that the respiratory and gastrointestinal systems were most affected and co-trimoxazole was the most prescribed antibiotic children despite being on ART. These findings suggest that caution should be applied when prescribing antibiotics.
**Order of Authors:**	Jonathan Gwasupika, MBcHB, MSc
	Davidson H Hamer
	Victor Daka
	Ephraim Chikwanda
	David Mwakazanga
	Ruth L Mfune
	Choolwe Jacobs
**Opposed Reviewers:**	
**Additional Information:**	
**Question**	**Response**
**Financial Disclosure**<br><br>Enter a financial disclosure statement that describes the sources of funding for the work included in this submission. Review	The author(s) received no specific funding for this work.

the submission guidelines for detailed requirements. View published research articles from *PLOS ONE* for specific examples.<br><br>This statement is required for submission and **will appear in the published article** if the submission is accepted. Please make sure it is accurate.<br><br>**Funded studies**<br>Enter a statement with the following details:<br>• Initials of the authors who received each award<br>• Grant numbers awarded to each author<br>• The full name of each funder<br>• URL of each funder website<br>• Did the sponsors or funders play any role in the study design, data collection and analysis, decision to publish, or preparation of the manuscript?<br><br>Did you receive funding for this work?	
**Competing Interests**<br><br>Use the instructions below to enter a competing interest statement for this submission. On behalf of all authors, disclose any competing interests that could be perceived to bias this work—acknowledging all financial support and any other relevant financial or non-financial competing interests.<br><br>This statement is required for submission and **will appear in the published article** if the submission is accepted. Please make sure it is accurate and that any funding sources listed in your Funding Information later in the submission form are also declared in your Financial Disclosure statement.<br><br>View published research articles from *PLOS ONE* for specific examples.	The authors declare that no competing interests exists

**NO authors have competing interests**<br><br>Enter: *The authors have declared that no competing interests exist.*<br><br>**Authors with competing interests**<br><br>Enter competing interest details beginning with this statement:<br><br>*I have read the journal's policy and the authors of this manuscript have the following competing interests: [insert competing interests here]*<br><br>* typeset	
**Ethics Statement**<br><br>Enter an ethics statement for this submission. This statement is required if the study involved:<br><br>• Human participants<br>• Human specimens or tissue<br>• Vertebrate animals or cephalopods<br>• Vertebrate embryos or tissues<br>• Field research<br><br>Write "N/A" if the submission does not require an ethics statement.<br><br>General guidance is provided below. Consult the submission guidelines for detailed instructions. **Make sure that all information entered here is included in the Methods section of the manuscript.**	Ethical approval to carry out the study was obtained from the TDRC Research Ethics Committee (IRB registration number 00002911). Permission to carry out the study and access to patient information was obtained from the Copperbelt Provincial Health Office (CPHO) and ADCH management. Confidentiality of patient information was adhered to and data were de-identified prior to analysis. Informed consent was not obtained from any individual as this was a retrospective chart review.

Powered by Editorial Manager® and ProduXion Manager® from Aries Systems Corporation

**Format for specific study types**<br><br>**Human Subject Research (involving human participants and/or tissue)**<br>• Give the name of the institutional review board or ethics committee that approved the study<br>• Include the approval number and/or a statement indicating approval of this research<br>• Indicate the form of consent obtained (written/oral) or the reason that consent was not obtained (e.g. the data were analyzed anonymously)<br><br>**Animal Research (involving vertebrate animals, embryos or tissues)**<br>• Provide the name of the Institutional Animal Care and Use Committee (IACUC) or other relevant ethics board that reviewed the study protocol, and indicate whether they approved this research or granted a formal waiver of ethical approval<br>• Include an approval number if one was obtained<br>• If the study involved *non-human primates*, add *additional details* about animal welfare and steps taken to ameliorate suffering<br>• If anesthesia, euthanasia, or any kind of animal sacrifice is part of the study, include briefly which substances and/or methods were applied<br><br>**Field Research**<br><br>Include the following details if this study involves the collection of plant, animal, or other materials from a natural setting:<br>• Field permit number<br>• Name of the institution or relevant body that granted permission	
**Data Availability**<br><br>Authors are required to make all data underlying the findings described fully available, without restriction, and from the time of publication. PLOS allows rare exceptions to address legal and ethical concerns. See the PLOS Data Policy and FAQ for detailed information.	Yes - all data are fully available without restriction

Powered by Editorial Manager® and ProduXion Manager® from Aries Systems Corporation

A Data Availability Statement describing where the data can be found is required at submission. Your answers to this question constitute the Data Availability Statement and **will be published in the article**, if accepted.<br><br>**Important:** Stating 'data available on request from the author' is not sufficient. If your data are only available upon request, select 'No' for the first question and explain your exceptional situation in the text box.<br><br>Do the authors confirm that all data underlying the findings described in their manuscript are fully available without restriction?	
**Describe where the data may be found in full sentences. If you are copying our sample text, replace any instances of XXX with the appropriate details.**<br><br>• If the data are **held or will be held in a public repository**, include URLs, accession numbers or DOIs. If this information will only be available after acceptance, indicate this by ticking the box below. For example: *All XXX files are available from the XXX database (accession number(s) XXX, XXX.).*<br>• If the data are all contained **within the manuscript and/or Supporting Information files**, enter the following: *All relevant data are within the manuscript and its Supporting Information files.*<br>• If neither of these applies but you are able to provide **details of access elsewhere**, with or without limitations, please do so. For example:<br><br>*Data cannot be shared publicly because of [XXX]. Data are available from the XXX Institutional Data Access / Ethics Committee (contact via XXX) for researchers who meet the criteria for access to confidential data.*<br><br>*The data underlying the results presented in the study are available from (include the name of the third party*	The data underlying the findings from this study are within the manuscript and its supporting information files.

and contact information or URL).<br>• This text is appropriate if the data are owned by a third party and authors do not have permission to share the data.<br><br>* typeset	
Additional data availability information:	

Powered by Editorial Manager® and ProduXion Manager® from Aries Systems Corporation

Public Health Department

Copperbelt University

P.O Box 71191

Ndola

4th January, 2024

The Editor

PLOS One

Dear Editor,

RE: Submission of original research for publication

The above subject refers.

I am hereby submitting a paper entitled '**Common clinical presentations associated with antibiotic use in HIV-infected children at a large referral Children's Hospital in Northern Zambia'.** The paper investigates prescribing patterns of antimicrobials in a paediatric HIV positive population at a paediatric hospital in Zambia. It is the author's view that this work will be of interest to the readers of your journal and other researchers keen on antinicobial resistance and HIV medicine and will add key knowledge to the body of knowledge pertaining to antimicrobial resistance and anti-microbial stewardship in Zambia and similar settings. This study also builds on previous work that looked at the morbidities among HIV positive children in Lusaka and focuses on antimicrobial resistance with implications for antimicrobial stewardship programs.

The authors declare that they have no competing interests and have approved the submission of this paper to your journal. This work has not been published or submitted for publication elsewhere.

Yours Sincerely,

Victor Daka- Corresponding author

Manuscript

Click here to access/download;Manuscript;Manuscript_Antibiotic use among

Common clinical presentations associated with antibiotic use in HIV-infected children at a large referral Children's Hospital in Northern Zambia.

Jonathan Gwasupika[1,2,3], Davidson H Hamer[3,4], Victor Daka[5], Ephraim Chikwanda[6], David Mwakazanga[7], Ruth L. Mfune[5], Choolwe Jacobs[2]

1. Department of Clinical Sciences, Tropical Diseases Research Centre, Ndola, Zambia

2. Department of Epidemiology and Biostatistics, School of Public Health, University of Zambia, Lusaka, Zambia

3. Department of Global Health, School of Public Health, Boston University, Boston, MA, USA

4. Department of Medicine, Section of Infectious Diseases, Boston University Chobanian & Avedisian School of Medicine, Boston, MA, USA

5. Public Health Department, School of Medicine, Copperbelt University, Ndola, Zambia

6. Department of Biomedical Sciences, Tropical Diseases Research Centre, Ndola, Zambia

7. Department of Public Health, Tropical Diseases Research Centre, Ndola, Zambia

***Corresponding author**: Victor Daka, Public Health Department, School of Medicine, Copperbelt University, Ndola, Zambia email: dakavictorm@gmail.com

Phone: +260975658073

Short running title: Clinical presentations associated with antibiotic use in HIV-infected children in Zambia

Abstract

Background

Children with HIV infection are disproportionately susceptible to bacterial infections. Despite the availability of anti-bacterial agents to manage HIV children with bacterial infections, the indiscriminate administration of antibiotics still occurs at health facilities which could lead to antimicrobial resistance.

Objectives

This study aimed to determine common antibiotics administered and associated clinical presentation in children at Arthur Davison Children's Hospital (ADCH) ART (antiretroviral therapy) clinic in Ndola, Zambia.

Methods

This was a retrospective analysis of prescribing patterns of routinely collected data at ADCH from 5th October to 20th November, 2023. Complete enumeration of files for all children attending ART care at ADCH from January 2011 to October 2022 was conducted. Children diagnosed with HIV before the age of 5, actively attending ART clinic identified by SmartCare software and who had taken ART for 3 months were eligible.

Results

There were 132 children included in the study and of these, 37.9% (50/132) presented with symptomatic complaints with the most common clinical presentation being cough (70%) followed by fever (34%) and diarrhoea (30%). A larger proportion of children were on abacavir/lamivudine/dolutegravir (ABC/3TC/DTG) (62.1%) for the ART regimen of choice. ~~Child~~ taking the abacavir/lamivudine/dolutegravir (ABC/3TC/DTG) regimen more frequently had complaints (48.8%) than those on TAF/3TC/DTG (21.0%) or TDF/3TC/DTG regimen (18.2%) (p=0.006).

Conclusions

Common clinical presentations showed that the respiratory and gastrointestinal systems were most affected and co-trimoxazole was the most prescribed antibiotic children despite being on ART. These findings suggest that caution should be applied when prescribing antibiotics.

Keywords: antibiotics, ART, HIV, clinical presentation

Introduction

The global fight against the human immunodeficiency virus (HIV) in children has changed since the availability of antiretroviral therapy (ART) [1]. This has been augmented by the implementation of all pregnant women testing positive to HIV being initiated on ART [2]. The improved access to ART in sub-Sahara Africa (SSA) has resulted in a decline in HIV related morbidity and mortality in children and adolescents [3,4]. The HIV paediatric program in Zambia has recorded a sustained progress with over 64% of children living with HIV accessing ART by the end of 2017 [5]. Despite the many achievements made in ART treatment for children,[6] some children present with opportunistic infections such as pneumonia and tuberculosis that are associated with immune impairment in uncontrolled HIV infection [7–10].

Children infected with HIV are at a greater risk of acquiring opportunistic infections [11] with respiratory tract infections (RTI) and pneumonia being the most common whether a child is on co-trimoxazole prophylaxis or not [12]. There has been an increase in the usage of antibiotics for prophylaxis or treatment in HIV infected children presenting with RTI or gastrointestinal symptoms [13]. Antibiotic use in this manner, if there is not a specific indication for antimicrobial therapy, poses a risk for the development of antibiotic resistance especially to commonly, cheap and easily accessible antibiotics [14]. This may further cause a challenge in treating infections among patients with immunosuppressive condition such as children infected with HIV [15]. The effectiveness of ART is mainly monitored clinically by assessing if a

child has presented with any symptom while on treatment [16,17]. This study was aimed at determining the most common clinical presentations and the associated antibiotics used among HIV infected children on ART at Arthur Davison's Children Hospital ART clinic.

Materials and Methods

Study design and site

This was a retrospective cross-sectional study of children infected with HIV seeking care at the ART outpatient clinic at Arthur Davison's Children Hospital (ADCH) from January 2011 to October 2022. ADCH is the largest tertiary specialised paediatric hospital in northern Zambia, and it is the referral centre for specialised HIV care in this region. As of October 2022, about 2600 HIV infected children were being followed at ADCH ART clinic with about 35 patients reviewed on a daily basis.

Study population and eligibility criteria

The study population was made up of all children attending ART care at ADCH from January 2011 to October 2022. A total of 321 file identification numbers were generated in SmartCare, an electronic health record management system [18] for all children who were attending ART clinic and files were reviewed. Complete enumeration of 132 files for all children meeting the eligibility criteria was done. Children who were diagnosed with HIV before the age of 5, actively attending ART clinic identified by SmartCare, and had taken ART for 3 months were included in the study. Children whose file was not present at the time of the study were excluded.

Data collection

Data were collected from files selected using the file selection procedure in SmartCare for active children from 5th October to 20th November, 2023. A file is created for every HIV infected child being seen at ADCH ART clinic and linked to care by SmartCare. A unique identification code is generated in smart-care which is then recorded on the file. File numbers for all children who were 5 years and below at time of HIV diagnosis were generated in SmartCare. Data collected from patient files were; age at HIV diagnosis, ART regimen, sex, presenting complaint 3 months after ART initiation and prescribed antibiotics.

Data Management and Analysis

Data were directly entered from the patient files into two password protected electronically structured data entry tools in kobo-collect. Each patient file was entered by two independent data entry persons. The resulting databases were then exported to Microsoft Excel and converted to STATA (STATA Corp., College Station, Texas, USA) software, version 16 SE. Codes were written in STATA comparing the data values between the two databases. Differences were resolved with reference to the source patient files.

We presented proportions of antibiotics for presenting complaints in HIV infected children seen at ADCH ART clinic respectively using a pie chart. Sex of the patients was described using frequencies and percentages. Age was presented as a continuous variable and described using the median and interquartile range (IQR). To test for

differences on whether a child presented with complaints 3 months after ART initiation, the Fishers exact test was used. For continuous normally distributed variables, the t-test was used. STATA software, version 16 SE (STATA Corp., College Station, Texas, USA) was used for analysis. A p-value less than 0.05 was considered statistically significant at a confidence interval of 95% (95% CI).

Ethical Approval

Ethical approval to carry out the study was obtained from the TDRC Research Ethics Committee (IRB registration number 00002911). Permission to carry out the study and access to patient information was obtained from the Copperbelt Provincial Health Office (CPHO) and ADCH management. Confidentiality of patient information was adhered to and data were de-identified prior to analysis. Informed consent was not obtained from any individual as this was a retrospective chart review and therefore waived in its approval by the research ethics committee.

Results

From a total of 321 children who were actively attending ART care at ADCH, 132 (41.1%) children met the inclusion criteria and were included in the study. Out of 132 children, 37.9% (50/132) presented with complaints (Table 1). The most common clinical presentation was cough (70%) followed by fever (34%) and diarrhoea (30%). There were slightly more males [51.5% (68/132)] than females. The minimum age of a child at HIV diagnosis was 1 month with the maximum being 60 months of age over the period 2011 to 2022. The median age at HIV diagnosis was 20 months (IQR 10 – 35 months). A larger proportion of children was noted to be on abacavir/lamivudine/dolutegravir (ABC/3TC/DTG) (62.1%) followed by tenofovir alafenamide/lamivudine/dolutegravir (TAF/3TC/DTG) (28.8%) for the ART regimen of choice (Table 1).

Table 1: Characteristics of children attending ART clinic, Ndola, Zambia, 2011 to 2022.

Variable	Category	Frequency (N)	Percentage (%)
Age at HIV diagnosis (in months)	Median (IQR)	20 (10.8 – 35.0)	
Weight (kg)	Mean (SD)	10.3 (5.1)	
Height (cm)	Median (IQR)	75.8 (62.8 – 91.0)	
Sex			
	Female	64	48.5
	Male	68	51.5
ART regimen			

	ABC/3TC/DTG	82	62.1
	TAF/3TC/DTG	38	28.8
	TDF/3TC/DTG	11	8.3
Complaint			
	No	82	62.1
	Yes	50	37.9
Presenting complaints			
	Fever	17	34
	Cough	35	70
	Diarrhoea	15	30
	Vomiting	3	6
	Appetite loss	6	12

HIV = Human immunodeficiency virus, IQR = interquartile range, SD = standard deviation, ABC = abacavir, 3TC = lamivudine, DTG = dolutegravir, ZDV = zidovudine, TAF = tenofovir alafenamide, TDF = tenofovir disoproxil

There was no difference between female children that presented for antiretroviral therapy (ART) care with complaints 3 months after ART initiation and male children (p=0.421) (Table 2). The mean age of children at HIV diagnosis was ~~more~~ among children with no complaints (25.2 months) than among children with complaints (20.9 months). It was noted that children more frequently presented with complaints when on ABC/3TC/DTG regimen (48.8%) compared to those on TAF/3TC/DTG (21.0%) or TDF/3TC/DTG regimen (18.2%) (p=0.006).

Table 2: Cross tabulation of factors in children presenting with complaints, Ndola, Zambia 2011 to 2022

Variable		No complaint	With complaint	p Value
Age at HIV diagnosis Mean (SD)		25.2 (17.3)	20.9 (12.4)	0.130[T]
Height (cm) Mean (SD)		69.0 (40.2)	63.7 (33.5)	0.431[T]
Weight (Kg) Mean (SD)		10.9 (5.6)	9.5 (3.9)	0.128[T]
Sex	Female	42 (65.6%)	22 (34.4%)	
	Male	40 (58.8%)	28 (41.2%)	0.421[P]
Regimen	ABC/3TC/DTG	42 (51.2%)	40 (48.8%)	
	TAF/3TC/DTG	30 (79.0%)	8 (21.0%)	
	TDF/3TC/DTG	9 (81.8%)	2 (18.2%)	0.006[F]

About 60% of children that presented with complaints were prescribed antibiotics. Co-trimoxazole was prescribed in 38% of children. Erythromycin and cephalexin were the least prescribed and type of antibiotic prescribed was missing in 40% of children (Figure 1).

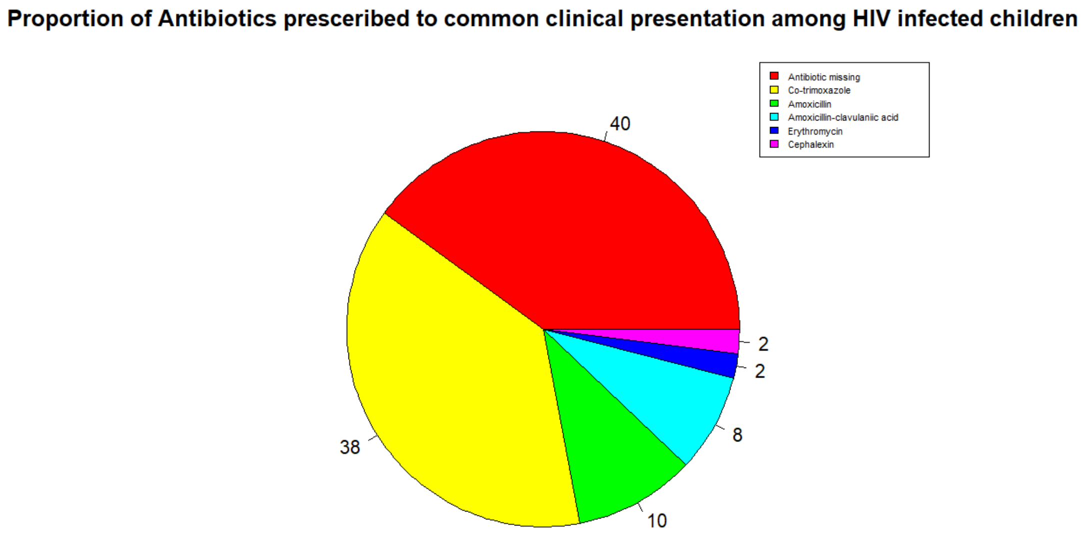

Figure 1: Prescribed antibiotics in children presenting with complaints, Ndola, Zambia, 2011 to 2022

Discussion

Our study found that 37.9% of the children presented with complaints and among these, cough was the most commonly reported complaint (70%) followed by fever (34%) and diarrhoea (30%). Clinically, children have an insidious onset of non-productive cough and most often the temperature would be normal common for respiratory tract infection caused by viruses [19]. A study in Malawi showed that about 37.5% presented with cough as one of the common presenting complaints [20], which was similar to our finding, even though, the proportion was different. Coughing was most prominent due to the fact that respiratory infections are the most frequently diagnosed opportunistic infections in HIV patients [21]. Administration of paracetamol in children who may have had a fever prior to seeking ART care may have contributed to the low proportion of fever as a presenting complaint in our study. A study in Nigeria showed different findings from our study, as fever was common (75%) followed by cough among HIV infected children [22]. Another study in India showed that about 43.6% presented with a cough and 50% presented with a fever suggesting that fever is quite common that cough [23], which was also different from our study findings. However, the differences would have been the timing of the two studies (e.g., in relation to seasonal respiratory viral infections), sample size difference, and geographical location differences.

About 30% of children seeking ART care presented with diarrhoea which was noted to be the third among the common presenting complaints. Prior to ART, diarrhoea

was reported in up to 90% of HIV-positive children residing in low-income settings and in 40 to 80% of those living in high-income countries [24]. Diarrhoeal disease has been known to affect 40% to 80% HIV infected persons and is associated with high mortality rates in sub-Saharan Africa [25]. Thus, the low proportion of patient presenting with diarrhoea found in this study could be attributed to administration of co-trimoxazole given in HIV infected people to prevent opportunistic infections [26]. The other contributing factor could be improved access to ART and HIV care [27].

About 60% of children presenting with common complaints were administered antibiotics. Various antibiotics are used to treat underlying conditions in HIV patients. From the above findings, co-trimoxazole (38%) being the most used, followed by amoxicillin (10%), cephalexin (8%), erythromycin (2%) and amoxicillin-clavulanic acid (2%). Co-trimoxazole prophylaxis is recommended for children born to women with HIV to protect those who acquire HIV from opportunistic infections as recommendation by WHO [28]. As a result, resistant patterns on co-trimoxazole are being observed. These findings are consistent with an effect of co-trimoxazole on resistance genes due to persistent antibiotic selection pressure and this is in agreement with a prior report from Lusaka that found modestly increased co-trimoxazole and clindamycin resistance among HIV-exposed infants receiving co-trimoxazole [29]. However, the background prevalence of co-trimoxazole resistance is extremely high in these settings. In Botswana, over 60% of infants at 2–4 weeks of age already had co-trimoxazole-resistant *Escherichia coli* isolates. Although the Botswana study found

some evidence of increased amoxicillin resistance which is used as a first-line treatment for common childhood infections, the baseline prevalence of amoxicillin resistance differed between arms in the few children studied [30]. A study in South Africa showed that co-trimoxazole led to alterations in the infant gut microbiome but did not promote major dysbiosis, since microbial taxa and functional pathways were not substantially different between infants receiving or not receiving co-trimoxazole at 4 and 6 months [31]. These findings suggest that caution should be applied when prescribing antibiotics.

Prescribers should be encouraged to adopt a rational use of antibiotics to reduce unnecessary prescriptions. This study had some limitations including the lack of data on CD4 and viral load to correlate with type of regimen children were on, analysis was done on secondary collected data thus there was minimal control on data such as knowing if the child was on co-trimoxazole prophylaxis or not as there was no documentation. A prospective study with more variables is recommended with data on HIV status (CD4 count, viral load) and ideally bacteriological data with antibiotic susceptibility patterns.

Funding

This study received no specific funding for the conduct of the study. However, JG is supported by Fogarty International Center and National Institute of Mental Health, of the National Institutes of Health under a fellowship under Award Number D43 TW010543. The content of this manuscript is solely the responsibility of the authors and does not necessarily represent the official views of the National Institutes of Health.

Transparency Declarations

None to declare

References

1. Huson MAM, Grobusch MP, van der Poll T. The effect of HIV infection on the host response to bacterial sepsis. Lancet Infect Dis. 2015;15(1):95–108.

2. Mutanga JN, Mutembo S, Ezeamama AE, Song X, Fubisha RC, Mutesu-Kapembwa K, Sialondwe D, Simuchembu B, Chinyonga J, Thuma PE, Whalen CC. Long-term survival outcomes of HIV infected children receiving antiretroviral therapy: An observational study from Zambia (2003-2015). BMC Public Health. 2019 Jan;19(1).

3. Makadzange AT, Higgins-Biddle M, Chimukangara B, Birri R, Gordon M, Mahlanza T, McHugh G, Van Dijk JH, Bwakura-Dangarembizi M, Ndung'u T, Masimirembwa C, Phelps B, Amzel A, Ojikutu BO, Walker BD, Ndhlovu CE. Clinical, Virologic, Immunologic outcomes and emerging HIV Drug resistance patterns in children and adolescents in public ART Care in Zimbabwe. PLoS One. 2015 Dec;10(12).

4. Taylor G. Sexually transmitted infections: Rolling out HIV antiretroviral therapy in sub-Saharan Africa: 2003–2017. Canada Commun Dis Rep [Internet]. 2018 Feb 2 [cited 2023 Mar 9];44(2):68. Available from: /pmc/articles/PMC5864409/

5. ZAMBIA POPULATION-BASED HIV IMPACT ASSESSMENT ZAMPHIA 2016.

6. Natukunda J, Kirabira P, Ong KIC, Shibanuma A, Jimba M. Virologic failure in

HIV-positive adolescents with perfect adherence in Uganda: a cross-sectional study. Trop Med Health. 2019 Jan;47(1):8.

7. Birhan TY, Gezie LD, Teshome DF, Sisay MM. Predictors of CD4 count changes over time among children who initiated highly active antiretroviral therapy in Ethiopia. Trop Med Health. 2020 May;48(1):1–8.

8. Diress G, Dagne S, Alemnew B, Adane S, Addisu A. Viral Load Suppression after Enhanced Adherence Counseling and Its Predictors among High Viral Load HIV Seropositive People in North Wollo Zone Public Hospitals, Northeast Ethiopia, 2019: Retrospective Cohort Study. AIDS Res Treat. 2020;2020.

9. Lawn SD, Meintjes G, McIlleron H, Harries AD, Wood R. Management of HIV-associated tuberculosis in resource-limited settings: A state-of-the-art review. BMC Med [Internet]. 2013 Dec 2 [cited 2023 Oct 8];11(1):1–16. Available from: https://bmcmedicine.biomedcentral.com/articles/10.1186/1741-7015-11-253

10. Sharma SK, Soneja M. HIV & immune reconstitution inflammatory syndrome (IRIS). Indian J Med Res. 2011;134(6):866.

11. Marbou WJT, Kuete V. Bacterial resistance and immunological profiles in HIV-infected and non-infected patients at Mbouda AD LUCEM Hospital in Cameroon. J Infect Public Health. 2017 May;10(3):269–76.

12. Jaspan HB, Huang LC, Cotton MF, Whitelaw A, Myer L. Bacterial disease and

antimicrobial susceptibility patterns in HIV-infected, hospitalized children: a retrospective cohort study. PLoS One [Internet]. 2008 Sep 24 [cited 2023 Oct 2];3(9). Available from: https://pubmed.ncbi.nlm.nih.gov/18813340/

13. Olaru ID, Tacconelli E, Yeung S, Ferrand RA, Stabler RA, Hopkins H, Aiken AM, Kranzer K. The association between antimicrobial resistance and HIV infection: a systematic review and meta-analysis. Clin Microbiol Infect [Internet]. 2021 Jun 1 [cited 2023 Oct 2];27(6):846–53. Available from: https://pubmed.ncbi.nlm.nih.gov/33813126/

14. Faiela C, Sevene E. Antibiotic prescription for HIV-positive patients in primary health care in Mozambique: A cross-sectional study. South African J Infect Dis. 2022 Feb;37(1).

15. Ngalani OJT, Mbaveng AT, Marbou WJT, Ngai RY, Kuete V. Antibiotic Resistance of Enteric Bacteria in HIV-Infected Patients at the Banka Ad-Lucem Hospital, West Region of Cameroon. Can J Infect Dis Med Microbiol. 2019;2019.

16. Fenta DA, Wube TB, Nuru MM. Long-Term Immunological and Virological Outcomes in Children Receiving Highly Active Antiretroviral Therapy at Hawassa University College of Medicine and Health Sciences, Southern Ethiopia. J Immunol Res. 2021;2021.

17. Mega TA, Usamo FB, Negera GZ. Abacavir versus Zidovudine-based

regimens for treatment of HIV-infected children in resource limited settings: A retrospective cohort study. BMC Pediatr. 2020 Mar;20(1):1–9.

18. Kaumba PC. Factors affecting the implementation of the SmartCare EHR system in Zambia. Soc Sci Humanit Open. 2023 Jan 1;7(1):100399.

19. Githinji LN, Mahtab S, Zühlke L, Lawrenson J, Myer L, Gray D, Zar H. Cardiopulmonary dysfunction in perinatally HIV-infected South African adolescents on antiretroviral therapy: baseline findings from the Cape Town Adolescent Antiretroviral Cohort. J Int AIDS Soc. 2019 Jul;22(7).

20. Mwalukomo T, Rylance SJ, Webb EL, Anderson S, O'Hare B, Van Oosterhout JJ, Ferrand RA, Corbett EL, Rylance J. Clinical Characteristics and Lung Function in Older Children Vertically Infected With Human Immunodeficiency Virus in Malawi. J Pediatric Infect Dis Soc [Internet]. 2016 Jun 10 [cited 2023 Nov 20];5(2):162–9. Available from: https://pubmed.ncbi.nlm.nih.gov/26407277/

21. Chiappini E, Bestetti M, Masi S, Paba T, Venturini E, Galli L. Discomfort relief after paracetamol administration in febrile children admitted to a third level paediatric emergency department. Front Pediatr. 2023 Mar;11:1075449.

22. Ogunbosi BO, Oladokun RE, Brown BJ, Osinusi KI. Prevalence and clinical pattern of paediatric HIV infection at the University College Hospital, Ibadan, Nigeria: a prospective cross-sectional study. Ital J Pediatr. 2011;37(1):29.

23. Dhaka G, Sherwal BL, Saxena S, Rai Y, Chandra J. Current trends in opportunistic infections in children living with HIV/AIDS in a tertiary care hospital in Northern India. Indian J Sex Transm Dis AIDS [Internet]. 2017 Jul 1 [cited 2023 Nov 21];38(2):142. Available from: /pmc/articles/PMC6085927/

24. Basile FW, Fedele MC, Vecchio A Lo. Gastrointestinal Diseases in Children Living with HIV. Microorganisms [Internet]. 2021 Aug 1 [cited 2023 Nov 20];9(8). Available from: /pmc/articles/PMC8398799/

25. Boniphace I, Omari M, Fred RS, Ferdinand M, Marcel T. HIV/AIDS Clinical Manifestations and their Implication for Patient Clinical Staging in Resource Limited Settings in Tanzania. Open AIDS J [Internet]. 2011 Mar 17 [cited 2023 Nov 20];5(1):9. Available from: /pmc/articles/PMC3103877/

26. Nigatu D, Dinegde NG, Sendo EG. Cotrimoxazole Prophylaxis Treatment Adherence and Associated Factors Among Human Immunodeficiency Virus (HIV) Exposed Children in Public Hospitals in Ilubabor Zone, Southwest Ethiopia, 2018. Open Public Health J. 2019 May;12(1):184–98.

27. Sütçü M, Acar M, Aktürk H, Hançerli Torun S, Beka H, Ağaçfidan A, Salman N, Somer A. Clinical Findings of Pediatric HIV Infection in a Tertiary Center in Turkey. Balkan Med J. 2017;34(3):239.

28. Homsy J, Dorsey G, Arinaitwe E, Wanzira H, Kakuru A, Bigira V, Muhindo M, Kamya MR, Sandison TG, Tappero JW. Protective efficacy of prolonged co-

trimoxazole prophylaxis in HIV-exposed children up to age 4 years for the prevention of malaria in Uganda: A randomised controlled open-label trial. Lancet Glob Heal [Internet]. 2014 Dec 1 [cited 2023 Nov 20];2(12):e727–36. Available from: http://www.thelancet.com/article/S2214109X14703298/fulltext

29. Mwenya DM, Charalambous BM, Phillips PPJ, Mwansa JCL, Batt SL, Nunn AJ, Walker S, Gibb DM, Gillespie SH. Impact of Cotrimoxazole on Carriage and Antibiotic Resistance of Streptococcus pneumoniae and Haemophilus influenzae in HIV-Infected Children in Zambia. Antimicrob Agents Chemother [Internet]. 2010 Sep [cited 2023 Nov 20];54(9):3756. Available from: /pmc/articles/PMC2934996/

30. Kitt E, Hayes M, Ballester L, Sewawa KB, Mulale U, Mazhani L, Arscott-Mills T, Coffin SE, Steenhoff AP. Assessing antibiotic utilization among pediatric patients inGaborone, Botswana. SAGE Open Med [Internet]. 2022 [cited 2023 Nov 20];10. Available from: /pmc/articles/PMC9939905/

31. D'Souza AW, Moodley-Govender E, Berla B, Kelkar T, Wang B, Sun X, Daniels B, Coutsoudis A, Trehan I, Dantas G. Cotrimoxazole Prophylaxis Increases Resistance Gene Prevalence and α-Diversity but Decreases β-Diversity in the Gut Microbiome of Human Immunodeficiency Virus–Exposed, Uninfected Infants. Clin Infect Dis [Internet]. 2020 Dec 31 [cited 2023 Nov 20];71(11):2858–68. Available from: https://dx.doi.org/10.1093/cid/ciz1186

Click here to access/download
Supporting Information
Dataset_paeds_data.csv

Click here to access/download
Other
Ethics Approval.pdf
