# Supplementary material for: Antibiotic prescription patterns and associated symptoms in children living with HIV at Arthur Davison Children’s Hospital in Ndola, Zambia
Source: PLoS One. 2025 Mar 5;20(3):e0316735. doi: 10.1371/journal.pone.0316735 (PMC11882085; doi:10.1371/journal.pone.0316735)
Supplement: Attachment — Submitted filename: Responses to Reviewers_PLOS.docx [file pone.0316735.s003.docx]

Copperbelt University

School of Medicine

Ndola

16^th^ April, 2024

The Editor,

PLOS One

Dear Editor

**RE: Submission of revised version of manuscript**

The above subject refers.

Reference is made to your letter dated 5^th^ March 2024 in which you raised some queries concerning our application. Below is our response to each of the issues raised.

Additionally, we have uploaded an unmarked manuscript, a marked manuscript, the rebuttal/response letter and an updated dataset which has taken into account the comment on the ART duration raised by reviewers.

Yours Sincerely,

Victor Daka

**Corresponding author**

**Responses to Editor and Reviewers**

1. The results section of the abstract should include explicit data that include the overall proportion of children who were prescribed antibacterial agents in addition to the symptoms. Then authors can thereafter, add information related to factors that were associated with antibacterial prescription. I emphasize that is misleading to report that cotrimoxazole was the most prescribed antibacterial agent yet the study did not collect data related to prior prophylactic use of the same.

Response: Thank you for this comment. Overall proportion of children that received antibacterials has been added in addition to symptoms. The authors note that a dose of 240mg once daily of Co-trimoxazole is given as prophylaxis and 480mg twice daily is prescribed as treatment. The Co-trimoxazole which was documented in files was prescribed as treatment.

2. The conclusion of the study should rely on the study findings and there are no such findings to indicate that prescribers were irrational or careless with antibacterial use.

Response: The comment is well noted and changes have been made.

3. Under the methods section: The authors should throw light on what they mean with a retrospective, cross-sectional study design. They should also explain why there was one year (Nov 2022 to Oct 2023) omitted from the data collection. They collected their data during the period starting Oct 2023, but then, they only collected data ending Oct 2022. Authors need to explain why they didn't include files until 3 months prior to data collection. This anomaly needs to be explained because it creates bias in their research.

Response: Thank you for the comment, the author meant review of routine collected data among HIV infected children was done as there was no prospective follow up of participants. Changes have since been made. Data were collected on children aged 5 years and below between 2011 and 2022. The earlier stated period from Nov 2022 to Oct 2023 was an error. Children were eligible regardless of duration of ART, however, data on presenting complaints were only captured if child had taken treatment for at least 6 months as this the period HIV viral load suppression is expected and not the earlier typo of 3 months and this has been updated.

4. Still under methods; authors need to explain how they treated data for children who suffered multiple episodes of complaints during the year considered for data collection (Nov 2022 to Oct 2023). Did they collect data for the multiple episodes or for the first episode, and reasons why.

Response: Analysis of data was performed on the first episode of presenting to the ART clinic with complaints despite multiple episodes of visits done by the child. The author did not consider other episodes of presentation as this required more complicated analysis of panel data

5. In order to support the selection of children who had been on ART for 3 months, authors need to include relevant literature in the background. Also prevalence data on causes of cough, fever and diarrhea should be presented for the study area. The is what authors should use this information as an indicator of the expected prevalence use of the different the antibacterial agents, and thereafter compare to their findings.

Response: This has been taken note of and relevant corrections have been made.

Reviewer #1: The authors have presented a well written manuscript with clear explanations . Some suggestions are as follows :-
1. For the title, maybe it would be better to write out the entire name of the hospital (Arthur Davison Children’s Hospital) instead of “large referral Children’s Hospital”.

Response: Changes to the title as been made as advised.

2. Line no 40: - Either mention the percentage (37.9%) or the number (50/132) but not both.

Response: This has been corrected as per suggestion

3. Line no 57: - It should be “sub-Saharan Africa”.

Response: This has been corrected as per suggestion

Reviewer #2: Title: The title "Common clinical presentations associated with antibiotic use..." is not in sync with the objective which seems to focus on "common antibiotics administered and associated clinical presentations".Throughout, there doesn't seem to be consistency in what the objective is focused on - is it the clinical presentations or the antibiotics?

Response: Thank you for the comment and authors agree. The title has been reviewed to suit the objective.

Method: it is not clear if the patients took ART for 3 months or for at least 3 months - please clarify. If it's for at least 3 months, please add the median ART duration.

Response: This has been updated to reflect the sampling approach that was employed considering the period when viral suppression takes place. Presenting complaints for Children attending ART were only entered once they were in care for at least 6 months. Therefore, children were included in the study regardless of their duration of ART.

Results: Were there any differences in age among the three regimen groups? The conclusion that cotrimoxazole was the most prescribed drug for the clinical presentations may be misleading as it has not been established if this was given for prophylaxis or not; and if or not the patients were already on cotrimoxazole before the onset of symptoms. The results also show that children taking ABC/3TC/DTG had more complaints compared to those taking TAF or TDF/3TC/DTG. What would be important to mention is the median age of these groups of children as TAF/TDF would not be prescribed in younger children and it is known that respiratory complaints are common in young children compared to older children and adults. Such details would therefore be of interest to the readers.

Response: Thank you for the comment and this was noted by the authors. Changes have been made as suggested to include median age of children on ART as well median age of children for each ART regimen.

Conclusion: the conclusion that "the findings suggest that caution should be applied when prescribing antibiotics" has no basis as the authors did not show any results that warrant the "caution"; although their literature review suggests development of resistance. I suggest rewording this section.

Response; This is well noted and conclusion has been reviewed and corrections have been made.
