# Supplementary material for: Antibiotic prescription patterns and associated symptoms in children living with HIV at Arthur Davison Children’s Hospital in Ndola, Zambia
Source: PLoS One. 2025 Mar 5;20(3):e0316735. doi: 10.1371/journal.pone.0316735 (PMC11882085; doi:10.1371/journal.pone.0316735)
Supplement: Attachment — Submitted filename: PLOS ONE Review - ADCH.docx [file pone.0316735.s004.docx]

Thank you for the opportunity to review this manuscript on the “Association of commonly prescribed antibiotics and clinical presentation in HIV-infected children at Arthur Davison Children’s Hospital in Ndola, Zambia.”

The efforts of the authors of this study are acknowledged and appreciated. Please find below, my comments and suggestions by line numbers.

| Line number | Comment |
| --- | --- |
| 39 - 42 | Please use either “symptoms” or “complaints” and not “symptomatic complaints”. By “clinical presentation”, do you mean these symptoms/complaints or the picture from a clinician’s perspective in which case you may need to use “diagnosis” or “diagnoses” as appropriate? |
| 47 | “… common complaints …” Is there a specific list of these common complaints? Or did you want to mention the 5 most common ones you found in your study? |
| 50 - 51 | “...showed that the respiratory and gastrointestinal systems were mostly affected …” Affected by what? |
| 52 | What calls for “prudent use of prescribed antibiotics”? |
| 53 (and 79) | Please rewrite “condition” here as “conditions” |
| 59 | Did you mean “The initiation of ART in all HIV-infected pregnant women has reduced the likelihood of mother-to-child transmission of the virus”? While this strategy has reduced the incidence of childhood HIV infection, these children belong to a defined population. |
| 61 | It would help if you did better at hyphenating certain word combinations representing one concept. For example, “sub-Saharan Africa” and “HIV-related”. I noticed that one of the reviewers has already brought your attention to this. |
| 66 - 69 | This sentence should have stopped at “tuberculosis”. |
| 70 - 71 | Please delete “pneumonia”. It is a type of respiratory tract infection (RTI) and should not be written as separate from RTIs. |
| 72 - 73 | This sentence seems to be free-floating. You may merge it with another sentence or delete it altogether. |
| 73 – 78 | You noted that antibiotics are prescribed for prophylaxis or treatment of infections in HIV-infected children. What therefore is your caution about the “manner” of antibiotic prescription? Are prophylaxis and treatment not “specific” indications for antimicrobial therapy? |
| 81 | Please change “if a child has presented …” to “if a child presents …” since “The effectiveness of ART ‘is’ mainly monitored …”. |
| 82 – 84 | I noticed that a reviewer already drew your attention to the wording of your objective. Did you aim to determine the indications for antimicrobial therapy in HIV-infected children at the ADCH or the commonly prescribed antibiotics in this target population? Please clarify and ensure consistency with your aim throughout your work. |
| 95 – 97 | Were the 2,600 children receiving care at the ART clinic sometime in October 2022 included in your study? This would not have mattered if your study period did not include October 2022. Can you please specify the date range of your study? |
| 104 | Remove the comma and add “and” in “… before the age of 5, actively attending ART clinic …” |
| 106 | Please change “Children whose file was not present at the time of the study were excluded.” to “Children whose files were not available at the time of the study were excluded.” |
| 110 | Add a hyphen i.e. “antibiotic-prescribing …” and choose either “records” or “files” not “record files”. |
| 117 | I noticed a reviewer has already called your attention to the dates here. Why November 2022? |
| 121 – 124 | Please rewrite this sentence to better convey the intelligent information inside it. |
| 126 – 127 | Please change to “Data was …”, “password-protected”, and “electronically-structured data entry tools …” |
| 133 | I find these “presenting complaints” too ambiguous. Did the children have antibiotics prescribed for something like heat rash for example?  Do you think it would read better if you left it at “antibiotics prescribed for prophylaxis and/or treatment of infections in HIV-positive children …?  I noticed that you later mentioned the prophylactic use of Co-trimoxazole in this target population. It would be better to include this in your introduction because it is a significant factor that may skew your findings because of its dual role. |
| 143 | Please write TDRC in full at first mention with the acronym in parentheses. You may use the acronym alone after that. |
| 145 – 146 | Did you obtain permission to conduct the study and have access to the records in one approval? Please state this clearly. |
| 152 | “From a total of 321 children who were actively attending ART care at ADCH …“ Please specify the period under review since not ALL their paediatric patients were included in your study and remove the “children” and leave it as “132 met …” |
| 160 | “The median duration for children on ART was 74 months …” Is this for ALL children on ART or those in your study? Please refrain from ambiguous statements. |
| 164 | “… of choice.” But how? Did these children get to choose their ART regimens? |
| 165 | Again, you need to specify the date range of your study in the day, month, and year.  Please change “appetite loss” to “loss of appetite”. It is easier to read it this way. |
| 187 – 190 | Did you mean “Antibiotics were prescribed for 60% of the patients with complaints. Co-trimoxazole was prescribed in 38% of them, while Erythromycin and Cephalexin were the least prescribed at 2% each. In 40% of these children, the antibiotic prescribed was not specified.”? |
| 199 – 201 | Please rewrite this sentence to better convey the intelligent information inside it. |
| 201 – 203 | Was the study in Malawi similar to yours? You should specify what their study was about before showing similar findings to yours. |
| 206 – 207 | “In children, most respiratory infections are due to viral causes with a few due to bacterial, parasitic, and fungal pathogens. |
| 207 | The studies with different findings from yours should be in another paragraph and not a continuation of those with findings similar to yours. |
| 208 | “… fever was the most common complaint (75%), followed by cough…“ |
| 209 | For all the studies to compare and contrast your findings to, please mention what they were about. “Another study in India” is not good enough. |
| 223 - 227 | Did you repeat your findings here? |
| 227 | Was it Erythromycin and Cephalexin or Erythromycin and Amoxicillin-Clavulanic acid that were the least prescribed? |
| 227 – 246 | Did you decide to assess antimicrobial resistance with a focus on Co-trimoxazole? Or did you add this for completeness since you assessed antibiotic use in this target group? Do you want to review your aim again and expand your objectives?  Again, always put a new finding/idea in a different paragraph, not a continuation of an ongoing discussion. |
| 247 | Please write your limitations as a different sub-section, not part of your discussion. |
| 254 | “This study received no specific funding for its conduct.” |
