# Supplementary material for: Antibiotic prescription patterns and associated symptoms in children living with HIV at Arthur Davison Children’s Hospital in Ndola, Zambia
Source: PLoS One. 2025 Mar 5;20(3):e0316735. doi: 10.1371/journal.pone.0316735 (PMC11882085; doi:10.1371/journal.pone.0316735)
Supplement: Attachment — Submitted filename: Response to reviewers.docx [file pone.0316735.s005.docx]

Copperbelt Univeristy

School of Medicine

Ndola

18^th^ October, 2024

The Editor

PlosOne

Dear Editor

RE: Response to Reviewers

The above subject refers.

Kindly note that we have attended to the comments from reviewers for the manuscript entitled “Association of commonly prescribed antibiotics and clinical presentation in HIV-infected children at Arthur Davison Children’s Hospital in Ndola, Zambia.” Our detailed response is given as individual responses to the reviewers below.

All authors have authorized the submission of the review.

Yours Sincerely

Dr. Victor Daka

Corresponding author

Thank you for the opportunity to review this manuscript on the “Association of commonly prescribed antibiotics and clinical presentation in HIV-infected children at Arthur Davison Children’s Hospital in Ndola, Zambia.”

The efforts of the authors of this study are acknowledged and appreciated. Please find below, my comments and suggestions by line numbers.

| Line number | Comment |
| --- | --- |
| 39 - 42 | Please use either “symptoms” or “complaints” and not “symptomatic complaints”. By “clinical presentation”, do you mean these symptoms/complaints or the picture from a clinician’s perspective in which case you may need to use “diagnosis” or “diagnoses” as appropriate?  Response: This has been noted and sentences have been revised |
| 47 | “… common complaints …” Is there a specific list of these common complaints? Or did you want to mention the 5 most common ones you found in your study?  Response: Common symptoms were mentioned based on the findings of the study |
| 50 - 51 | “...showed that the respiratory and gastrointestinal systems were mostly affected …” Affected by what?  Response: The statement was rephrased |
| 52 | What calls for “prudent use of prescribed antibiotics”?  Response: This statement was rewritten |
| 53 (and 79) | Please rewrite “condition” here as “conditions”  Response: Sentence has been rewritten to include conditions |
| 59 | Did you mean “The initiation of ART in all HIV-infected pregnant women has reduced the likelihood of mother-to-child transmission of the virus”? While this strategy has reduced the incidence of childhood HIV infection, these children belong to a defined population.  Response: The sentence in the introduction has been rephrased |
| 61 | It would help if you did better at hyphenating certain word combinations representing one concept. For example, “sub-Saharan Africa” and “HIV-related”. I noticed that one of the reviewers has already brought your attention to this.  Response: This is well noted |
| 66 - 69 | This sentence should have stopped at “tuberculosis”  Responses: Sentence has been rephrased. |
| 70 - 71 | Please delete “pneumonia”. It is a type of respiratory tract infection (RTI) and should not be written as separate from RTIs.  Response: This has been noted |
| 72 - 73 | This sentence seems to be free-floating. You may merge it with another sentence or delete it altogether.  Response: This is noted |
| 73 – 78 | You noted that antibiotics are prescribed for prophylaxis or treatment of infections in HIV-infected children. What therefore is your caution about the “manner” of antibiotic prescription? Are prophylaxis and treatment not “specific” indications for antimicrobial therapy?  Sentence: The statement has been paraphrased |
| 81 | Please change “if a child has presented …” to “if a child presents …” since “The effectiveness of ART ‘is’ mainly monitored …”.  Response: Change has been made |
| 82 – 84 | I noticed that a reviewer already drew your attention to the wording of your objective. Did you aim to determine the indications for antimicrobial therapy in HIV-infected children at the ADCH or the commonly prescribed antibiotics in this target population? Please clarify and ensure consistency with your aim throughout your work.  Response: The objective has been rephrased |
| 95 – 97 | Were the 2,600 children receiving care at the ART clinic sometime in October 2022 included in your study? This would not have mattered if your study period did not include October 2022. Can you please specify the date range of your study?  Response: 132 participants were selected from the total 2600 already in care from 2011 to October 2022 |
| 104 | Remove the comma and add “and” in “… before the age of 5, actively attending ART clinic …”  Response: Change have been made following the recommendation |
| 106 | Please change “Children whose file was not present at the time of the study were excluded.” to “Children whose files were not available at the time of the study were excluded.” |
| 110 | Add a hyphen i.e. “antibiotic-prescribing …” and choose either “records” or “files” not “record files”.  Response: This has been well noted and corrections have been made |
| 117 | I noticed a reviewer has already called your attention to the dates here. Why November 2022?  Response: November was a typo and necessary changes have been made |
| 121 – 124 | Please rewrite this sentence to better convey the intelligent information inside it. |
| 126 – 127 | Please change to “Data was …”, “password-protected”, and “electronically-structured data entry tools …”  Response: Sentence has been changed as recommended |
| 133 | I find these “presenting complaints” too ambiguous. Did the children have antibiotics prescribed for something like heat rash for example?  Do you think it would read better if you left it at “antibiotics prescribed for prophylaxis and/or treatment of infections in HIV-positive children …?  I noticed that you later mentioned the prophylactic use of Co-trimoxazole in this target population. It would be better to include this in your introduction because it is a significant factor that may skew your findings because of its dual role.  Response: The discussion section has been rewritten and the word symptom has been used to replace presenting complaint as recommended. It was an oversight to include statement on prophylaxis as this was not easy to ascertain. |
| 143 | Please write TDRC in full at first mention with the acronym in parentheses. You may use the acronym alone after that.  Response: TDRC has been written in full |
| 145 – 146 | Did you obtain permission to conduct the study and have access to the records in one approval? Please state this clearly.  Response: Waiver to conduct the study was sought from an ethical committee board and authority was obtained from the national research authority. Permission to use hospital files was obtained from the hospital management. Therefore, approvals for the study were sought at different levels |
| 152 | “From a total of 321 children who were actively attending ART care at ADCH …“ Please specify the period under review since not ALL their paediatric patients were included in your study and remove the “children” and leave it as “132 met …”  Response: The sentence has been rephrased according to the recommendation |
| 160 | “The median duration for children on ART was 74 months …” Is this for ALL children on ART or those in your study? Please refrain from ambiguous statements.  Response: The median age of 74 months calculated was for the sampled children in the study not for all children under care |
| 164 | “… of choice.” But how? Did these children get to choose their ART regimens?  Response: The sentence has been rephrased |
| 165 | Again, you need to specify the date range of your study in the day, month, and year.  Please change “appetite loss” to “loss of appetite”. It is easier to read it this way.  Response: This was noted and corrections were made |
| 187 – 190 | Did you mean “Antibiotics were prescribed for 60% of the patients with complaints. Co-trimoxazole was prescribed in 38% of them, while Erythromycin and Cephalexin were the least prescribed at 2% each. In 40% of these children, the antibiotic prescribed was not specified.”?  Response: In 40% of participants that presented with symptoms, antibiotics were not specified. The figure 3 has been redone to show the percentages better |
| 199 – 201 | Please rewrite this sentence to better convey the intelligent information inside it.  Response: Sentence has been rewritten |
| 201 – 203 | Was the study in Malawi similar to yours? You should specify what their study was about before showing similar findings to yours.  Response: The sentence was rephrased |
| 206 – 207 | “In children, most respiratory infections are due to viral causes with a few due to bacterial, parasitic, and fungal pathogens.  Response: This was well noted |
| 207 | The studies with different findings from yours should be in another paragraph and not a continuation of those with findings similar to yours.  Response: The discussion has been rewritten following recommendation |
| 208 | “… fever was the most common complaint (75%), followed by cough…“  Response: sentence has been re-written according to recommendation |
| 209 | For all the studies to compare and contrast your findings to, please mention what they were about. “Another study in India” is not good enough.  Response: This was well noted |
| 223 - 227 | Did you repeat your findings here?  Response: The paragraph section has been re-written |
| 227 | Was it Erythromycin and Cephalexin or Erythromycin and Amoxicillin-Clavulanic acid that were the least prescribed?  Response: Corrections have been made as the least prescribed were Cephalexin and Erythromycin |
| 227 – 246 | Did you decide to assess antimicrobial resistance with a focus on Co-trimoxazole? Or did you add this for completeness since you assessed antibiotic use in this target group? Do you want to review your aim again and expand your objectives?  Again, always put a new finding/idea in a different paragraph, not a continuation of an ongoing discussion.  Response: The discussion has been redone to suit the objective |
| 247 | Please write your limitations as a different sub-section, not part of your discussion.  Response: Limitations have been put as a separate sub-section |
| 254 | “This study received no specific funding for its conduct.”  Response: sentence has been changed according to recommendation |

Journal Requirements:

Please review your reference list to ensure that it is complete and correct. If you have cited papers that have been retracted, please include the rationale for doing so in the manuscript text, or remove these references and replace them with relevant current references. Any changes to the reference list should be mentioned in the rebuttal letter that accompanies your revised manuscript. If you need to cite a retracted article, indicate the article’s retracted status in the References list and also include a citation and full reference for the retraction notice.

Review Comments to the Author

Reviewer #1: The manuscript has addressed all the comments, suggestions and modifications provided by recommenders in the previous round of reviews. HIV disproportionately affects children when it comes to bacterial infections, and the usage of antibiotics is connected to antimicrobial resistance. The authors have aimed to understand how common antibiotics are associated with an unwanted outcome, and the research was based in a children's hospital in Zambia.

Reviewer #3: Thank you for inviting me to review the manuscript titled “Association of commonly prescribed antibiotics and clinical presentation in HIV-infected children at Arthur Davison Children’s Hospital in Ndola, Zambia”. The aim of this study was to determine the most common clinical presentations and the associated antibiotics used among HIV infected children on ART at Arthur Davison’s Children Hospital ART clinic.

Below are some suggestions that the authors may find helpful:

Abstract
• Line 23: A recommendation is for the authors to consider defining HIV before using the abbreviation in the abstract and the body of the manuscript.

Response: HIV has been written in full in the abstract and body of the manuscript.
• Lines 29-30: Antiretroviral therapy should come before the abbreviation (ART).

Response: This has been amended
• Lines 41-42: Would it be better to include the "(62.1%)" after “A larger proportion of children” on line 41, rather than the current location on line 42?

Responded: The percentage 62.1% has been put after the statement
• Lines 43-45: A recommended guideline is to define abbreviations upon first appearance in the text. The definition of ABC/3TC/DTG was included in the abstract before the abbreviations were used. A recommendation is to define TDF/3TC/DTG and TAF/3TC/DTG before using their respective abbreviations.

Response: Abbreviations for the regimen have been written in full
• Lines 50-54: The abstract has a captivating conclusion.

Response: The conclusion has been refined to be clear

Introduction
• Lines 75-76: The sentence appears incomplete, as a word or some words are missing. Please update as needed.

Response: Sentence has been updated

Methods
• Line 95-96: Makes mention of 2600 HIV infected children being followed at the ADCH ART clinic. It might be preferable to update the sentence to reflect the type of following that was done. A consideration could be to rephrase that portion of the sentence as “2600 HIV infected children were followed-up at ADCH ART”.

Response: The sentence has been rephrased
• Study population and eligibility criteria: After applying the inclusion criteria, how many participants were included in the study?

Response: The total number of children living with HIV that were being seen at the time of data collection were 2600, however the researcher screened 321 files of children and selected 132 that met the eligibility criteria.

• Data collection: The manuscript previously stated that the study population for this study were HIV positive children seeking care at ADCH ART from January 2011 to October 2022. The data collection section of the manuscript however makes mention of “record files for active children attending ART from 1 October to 30 November 2022 were assessed for eligibility.” Does this imply that the final participants considered for inclusion in the study were only for children attending ART from 1 October to 30 November 2022? How many children attended the ART clinic from October 1 to November 30, 2024, and how many met the inclusion criteria? Kindly clarify information about those included in the study. What was the final number of study participants?

Response: Thank you for the clarification, this could have been a typo as the period from which participants were to be selected was 1 January 2011 to 31 October 2022 and not 30 November 2022. The final number of study participants analysed was 132.
• Line 117: To prevent confusion on the part of the reader, it might be better to include the timeline for students attending the ART clinic as October 1st 2022 to November 30th 2022.

Response: Time lines have been included

Data management and analysis
• The authors noted “Mann Whitney ranksum test” as a nonparametric test used in the study analyses. Kindly cross-check the preferable way for writing Man Whitney test – if it should be Mann Whitney ranksum test, Mann Whitney rank sum test or Mann Whitney rank-sum test.

Response: Different authors have written test differently, The author for this manuscript came “Mann Whitney rank-sum test” as the preferred way.

Ethical approval
• Line 143: Please include the full meaning of TDRC before using the abbreviation.

Response: TDRC has been written in full

Results
• Line 152: It is confusing how the number of study participants reduced from the target population of 2,600 to 321. Please include details of the selection, inclusion criteria and the final number of study participants included in the study in the methods section of the manuscript.

Response: 2600 is the total number of children living with HIV in care, 321 was the number of files screened and the study analysed data for 132 participants
• Line 154: The percentage and number of males in the study as [51.5% (68/132)]. There is no need to include both the percentage and the fraction of the study population that are male. Only the percentage can be reported.

Response: This has been well noted
• Line 158: Same comment as for line 154. Using only the percentage should work well.

Response: This has been well noted
• Line 162: Consider changing “A larger proportion of children was noted” to “A larger proportion of children were noted”.

Response: This has been considered
• Table 1: Some percentages do not add up to 100%. Please cross-check the figures in the manuscript for accuracy.

Response: The percentages have been recalculated to ensure that they add up.
• Figure 1: Figure 1 is not labeled. Please label figure 1 based on the journal guidelines.

Response: Figure has been labelled
• Lines 180-182: The percentages in-text do not add up to 100%. Is some data missing or are the additional percentages not reported in-text?

Response: This has been clarified
• Table 2: Please cross check the P-value column of the table. Confirm the journal and citation requirements for how to write P-value. Superscripts “M”, “T”, “P” and “F” are noted on P-values in the table. A recommendation is to include a footnote that defines the abbreviations at the end of the table.

Response: Definitions of superscripts of the p values for the tests done have been written in full and included in the footnote of the table
• Lines 188-189: This sentence is a bit confusing. Consider revising.

Response: The sentence has been revised

Discussion
• Lines 199-201: This sentence is confusing. Please rephrase.

Response: Rephrasing of the sentence has been done
• Lines 207-208: The percentage of participants with fever from the Nigerian study were included. However, the percentage of those with cough was not included. What is the percentage of HIV infected children with cough in the Nigerian study? Please include the percentage of children with cough.

Response: The sentence has been rewritten to include percentage of cough
• Lines 211-213: The manuscript made mention of the timing of two studies. Providing more information about the timing of the two studies and other relevant information that could be attributable to the differences noted would be helpful to the reader.

Response: This has been noted and sentence has been rewritten following recommendation
• Line 214-215: The thought in this sentence is incomplete. Some words seem to be missing.

Response: The sentence has been reconstructed
• Line 218: A word seems to be missing on this line. Consider also adding a comma in the sentence.

Response: The sentence has been reconstructed
• Line 221: Would it be preferable to replace “given in HIV infected people to prevent opportunistic infections” with “prescribed to in HIV infected people to prevent opportunistic infection”?

Response: Replacement has been done
• Line 228: Replace “children born from HIV positive women” with “children born to HIV positive women”.

Response: Replacement has been done
• Lines 237-240: The manuscript made mention of information from a Botswana paper. It is however unclear what “resistance differed between the arms in the few children studied” noted on line 240 means. Please clarify this sentence.

Response: The sentence in the discussion has been rewritten to answer main objective
• Line 247: Does CD4 refer to CD4 cell count? If so, the authors can add the information for the purpose of clarity.

Response: This was supposed to read as CD4 percentage as it is the preferred way of presenting in children compared to CD count mainly used in adolescents and adults
• Line 250: Please provide more information about what “due to lack of documentation” means in the context of the sentence on lines 248-250.

Response: This could have been a typographical error and corrections have been made

Acknowledgement
• Lines 256-258: The last five words in the first sentence seem out of place. There could be a better way to phrase the sentence “JG would like to acknowledge the Fogarty International Center and National Institute of Mental Health, of the National Institutes of Health for supporting his post-doctoral fellowship under Award Number D43 TW010543 despite not funding this study.”

Response: Corrections have been made following recommendation

Minor comments
• The purpose of this study was to determine the most common clinical presentations and the associated antibiotics used among HIV infected children on ART at Arthur Davison’s Children Hospital ART clinic. The manuscript discussed major clinical presentations. For comparison, a recommendation is to include information about the most common and the other clinical presentations of the study population.

Response: Thank you for the comment and this has been well noted
• Cross-check the manuscript for tenses, punctuation, especially the inclusion of commas in complex sentences and spaces after full-stops.

Response: Well noted and corrections have been made
• Tables: Cross-check the tables to confirm that correct figures are reported, and the percentages add up to 100%. Some numbers in the tables were reported with one decimal place while others were rounded up to the nearest whole number. Is there a specific reason for this? A recommendation to allow for uniformity in the percentage reported in the tables is to report the percentages in one decimal place.

Response: Well noted and recommendations have been taken into consideration

Previous reviewer feedback follow-up:
3. Under the methods section: The authors should throw light on what they mean with a retrospective, cross-sectional study design. They should also explain why there was one year (Nov 2022 to Oct 2023) omitted from the data collection. They collected their data during the period starting Oct 2023, but then, they only collected data ending Oct 2022. Authors need to explain why they didn't include files until 3 months prior to data collection. This anomaly needs to be explained because it creates bias in their research.
• The authors have not fully responded to this feedback. The first part remains addressed in the manuscript.

Response: The study included children who were in care from January 2011 to October 2022. Necessary corrections have been made in the manuscript under the method section.
• Retrospective or prospective are often used in describing cohort studies. This is a cross-sectional study. A recommendation is for the authors to define the study as a cross-sectional study, while providing the details of the study in the methods section to reflect why no informed consent documents were required for the study.

Response: This has been noted

Conclusion: the conclusion that "the findings suggest that caution should be applied when prescribing antibiotics" has no basis as the authors did not show any results that warrant the "caution"; although their literature review suggests development of resistance. I suggest rewording this section.
• The sentence referenced in the previous feedback still features on lines 244-245. Please advise.

Response: Conclusion has been redone
