# Supplementary material for: Antibiotic prescription patterns and associated symptoms in children living with HIV at Arthur Davison Children’s Hospital in Ndola, Zambia
Source: PLoS One. 2025 Mar 5;20(3):e0316735. doi: 10.1371/journal.pone.0316735 (PMC11882085; doi:10.1371/journal.pone.0316735)
Supplement: Attachment — Submitted filename: Responses to Plos One.docx [file pone.0316735.s007.docx]

On the first line in the abstract section, please confirm if immune-deficiency virus (HIV) should be immunodeficiency virus (HIV). The first sentence in the introduction section defines HIV as immunodeficiency. Please advise.

Response: Correction has been made in the abstract to read immunodeficiency instead of immune-deficiency.

Lines 68 - 70: It could be preferable to write “Because of the greater risk of acquiring opportunistic infections, HIV-infected and exposed children are prescribed co-trimoxazole prophylaxis due to its antibacterial, antifungal and antiparasitic properties.” In place of “Because of the greater risk of acquiring opportunistic infections, HIV-infected and exposed children are prescribed on co-trimoxazole prophylaxis due to its antibacterial, antifungal and antiparasitic properties.”

Response: Thank you for the comment, changes have been made as advised.

Line 94: Define ADCH before using the abbreviation.

Response: ADCH has been defined as advised.

Methods: Some important information is missing in the methods section about the study population. Information provided in the methods section should be such that another researcher can reproduce the study if they follow the methods outlined in the manuscript using the same dataset used by the authors. How many children in total attended the ADCH outpatient clinic between January 11 and October 2022? I believe based on the inclusion criteria, a total of 321 files were included in the study. Information about the study population; and how based on the inclusion criteria, the final number of children included in the study was 321 should be clearly described.

Response: The study reviewed 321 files of children under care and after screening for eligibility criteria, 132 were included in the study for analysis

Line 204: Does this phrase “Antibiotics were specified in about by 60.0% of HIV-positive children…” mean that antibiotics were used by 60.0% of HIV-positive children…? If so, please update the manuscript to prevent confusion on the part of the reader.

Response: This has been corrected as advised.

Limitations: What are the other limitations of using secondary data relevant to this study?

Response: We have recast the limitations to add the possible effect of missing data.
